# Comparison of the impact of prolonged low-pressure and standard-pressure pneumoperitoneum on myocardial injury after robot-assisted surgery in the Trendelenburg position: study protocol for a randomized controlled trial

**DOI:** 10.1186/s13063-016-1609-5

**Published:** 2016-10-10

**Authors:** Xixue Zhang, Jionglin Wei, Xiaoxing Song, Yuhao Zhang, Weiqing Qian, Lu Sheng, Zhoujun Shen, Lvjun Yang, Rong Dong, Weidong Gu

**Affiliations:** 1 Department of Anesthesiology, Huadong Hospital Affiliated to Fudan University, Shanghai, China; 2 Department of Anesthesiology, Ruijin Hospital, Shanghai Jiaotong University School of Medicine, Shanghai, China; 3 Department of Urology, Huadong Hospital Affiliated to Fudan University, Shanghai, China; 4 Department of Urology, Ruijin Hospital, Shanghai Jiaotong University School of Medicine, Shanghai, China

**Keywords:** Low-pressure pneumoperitoneum, Standard-pressure pneumoperitoneum, Myocardial injury, Troponin T, Trendelenburg position, Robot-assisted urological surgery

## Abstract

**Background:**

Robot-assisted laparoscopic radical prostatectomy and robot-assisted radical cystectomy have gradually become the preferred choices for urologists as they allow surgeons to perform complex procedures more precisely and effectively. The pneumoperitoneum, which is normally applied in these surgeries to provide visual clarity and space to perform the procedure, may cause hemodynamic disturbance, potentially myocardial injury. Thus surgeons have recently considered opting for the low-pressure pneumoperitoneum to lower this negative impact.

Herein we describe a protocol for a clinical trial to compare the impact of prolonged low-pressure and standard-pressure pneumoperitoneum on myocardial injury after robot-assisted surgery.

**Methods/design:**

This study is designed to be a bicenter clinical trial. In total 280 patients scheduled to undergo robot-assisted laparoscopic radical prostatectomy or robot-assisted radical cystectomy will be enrolled and randomized into two groups, with standard- (12–16 mmHg) and low-pressure (7–10 mmHg) pneumoperitoneum, respectively. Troponin T will be measured as the primary endpoint to assess the extent of myocardial injury. Nt-proBNP and hemodynamic indexes will also be recorded for further analysis.

**Discussion:**

The significance of this study is emphasized by the fact that there are few studies that have focused on the impact of prolonged pneumoperitoneum on myocardial injury, which is relevant to postoperative mortality. We hope that the conclusions drawn from this study could provide reference and basis to the future of the pneumoperitoneum in clinical practice.

**Trial registration:**

Registered at https://www.clinicaltrials.gov with the Identifier NCT02600481 on November 5, 2015

**Electronic supplementary material:**

The online version of this article (doi:10.1186/s13063-016-1609-5) contains supplementary material, which is available to authorized users.

## Background

Robot-assisted surgery allows doctors to perform complex procedures with more precision, flexibility and control. As two major applications, robot-assisted laparoscopic radical prostatectomy (RALRP) and robot-assisted radical cystectomy (RARC) were firstly reported in 2000 [1–3] and 2003 [4], respectively. These procedures are rapidly gaining acceptance among urologists, as the robotic device offers the advantage of the minimally invasive laparoscopic approach but shortens the surgical learning curve [5, 6]. RALRP is now a choice of surgical treatment in most centers of excellence in the USA as well as in other parts of the world, which benefit from its superior visualization and magnification with respect to perioperative parameters [7]. In 2009, over 60,000 cases of RALRP were performed globally, accounting for 70 % of the total number of radical prostatectomies [8]. For RARC, its proportion in the USA increased from 0.6 % in 2004 to 12.8 % in 2010 [9, 10].

Standard-pressure pneumoperitoneum (SPP) is usually achieved by insufflating the abdominal cavity with CO_2,_ and an intra-abdominal pressure (IAP) of 12 to 16 mmHg is considered to be appropriate. SPP usually changes the IAP and systemic hemodynamics [11] causing oxidative [12], ischemia and reperfusion injury in various tissues [13]. Cardiac, pulmonary, intestinal [14], renal [15] and testicular [16] damage caused by SPP have been reported previously. When CO_2_ pneumoperitoneum is performed, the absorption of CO_2_ may lead to arrhythmias, mild acidemia and even severe acidemia; mean blood pressure, heart rate and pulmonary vascular resistance can also be increased [17]. RALRP and RARC are usually performed in the Trendelenburg position, with the patient positioned head-down at a specific angle (normally 15–30°). SSP in the Trendelenburg position causes a drop in left ventricular end-diastolic volume as measured by transthoracic echocardiography; it also affects diastolic function with a delay in deceleration time and isovolumetric relaxation time [18]. The pressure changes caused by pneumoperitoneum can decrease venous return, and subsequently affect cardiac output [19, 20]. All these hemodynamic disturbances may cause prolonged cardiac overload as well as myocardial oxygen imbalance, as the robot-assisted urological surgery usually lasts more than 3 h. Therefore, the potential myocardial injury thus caused is worth investigating.

To lower the impact of pneumoperitoneum on myocardial physiological status while providing appropriate space for operation, low-pressure pneumoperitoneum (LPP) at a range of 7–10 mmHg is considered optimum by surgeons. Numerous clinical trials have been done to compare LPP with SPP in laparoscopic cholecystectomy, the duration of which is often under 2 h [21]. A high IAP pneumoperitoneum was found to be associated with more fluctuations in hemodynamic parameters and increased peritoneal absorption of CO_2_ as compared to LPP in laparoscopic cholecystectomy [22]. Cardiac electrophysiological analysis showed that LPP results in lower sympathetic activation than SPP [23]. Prolongation of corrected QT dispersion (QTcd), which is associated with cardiovascular morbidity and mortality, was significantly higher in the high-pressure pneumoperitoneum group (15 mmHg) than in the LPP group (7 mmHg) [24]. In addition, a study focusing on solid intra-abdominal-organ flow suggested that increased intraperitoneal pressure substantially reduced the portal venous flow as compared with lower IAP [25]. A randomized controlled trial conducted by Kanwer et al. showed that LPP used in laparoscopic cholecystectomy benefited patients by lowering postoperative pain intensity [25, 26]. Hua et al. performed a meta-analysis in 2013 and showed that LPP was feasible and safe, resulted in reduced postoperative pain and nearly the same operative time as SPP [26, 27]. Prolonged robot-assisted surgery normally takes 4–5 h, sometimes 7–8 h, which is much longer than the time taken for cholecystectomy. Thus the outcome might be different, due to the different lengths of surgical time. So far, there are few studies concerning the comparison of LPP and SPP on myocardial injury after prolonged robot-assisted urological surgery. Postoperative myocardial injury after noncardiac surgery (MINS) has been proved to be an independent predictor of 30-day mortality [28]. An elevated troponin T (TnT) level after noncardiac surgery, irrespective of the presence of an ischemic feature, independently predicted 30-day mortality. A peak TnT level of 0.03 ng/mL or greater was used as diagnostic criterion for MINS in a cohort study reported in 2014 [29]. The N-terminal of the prohormone brain natriuretic peptide (Nt-proBNP) is an indicator for cardiac failure and is being used in routine tests in the emergency room [30]. It has been demonstrated that peak Nt-proBNP levels at peak IAP were correlated positively with indexes of cardiac afterload (systemic vascular resistance (SVR) and peripheral vascular resistance (PVR)). A strongly negative correlation between Nt-proBNP levels and cardiac output at peak IAP was also reported [31].

The present study was designed to compare the effects of prolonged LPP and SPP on myocardial injury after a relatively long period of surgery. The TnT level is chosen as a primary endpoint for the evaluation of myocardial injury as it has been shown to be a sensitive predictor of cardiac injury. Our working hypothesis is that LPP can result in improved myocardial outcome after robot-assisted urological surgery.

## Methods/design

### Study design

**Fig. 1:**
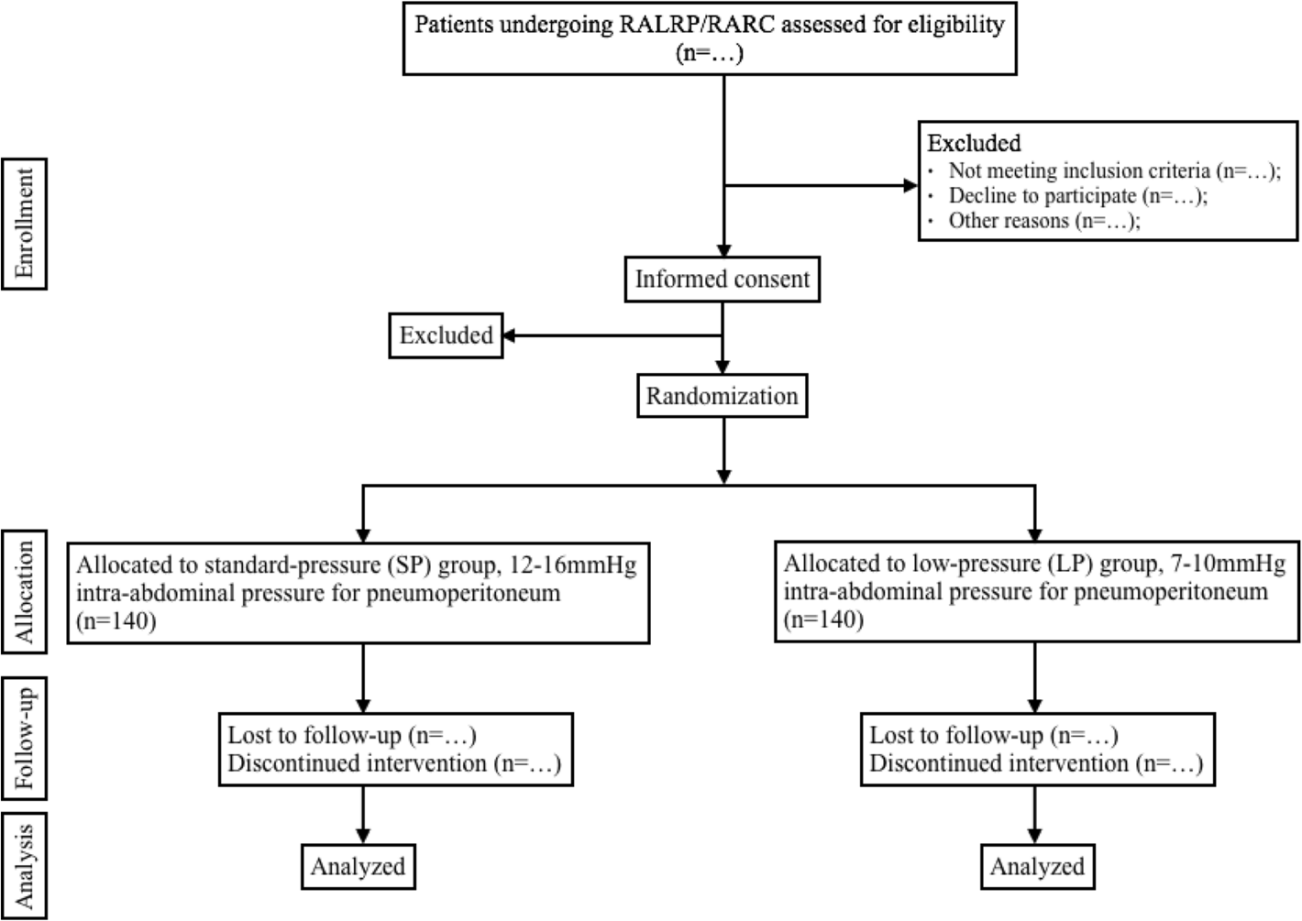
Study design and participant flow chart. *RALRP* robot-assisted laparoscopic radical prostatectomy, *RARC* robot-assisted radical cystectomy

This study is a prospective, bicenter, single-blinded, randomized controlled clinical trial to investigate the different effects of prolonged LPP and SPP on myocardial injury after RALRP and RARC (Fig. 1).

### Study population

Patients who are scheduled to undergo long-duration RALRP or RARC in the Huadong Hospital Affiliated to Fudan University and the Ruijin Hospital Shanghai Jiao Tong University School of Medicine, in line with the inclusion criteria, will be recruited. From the previous data and estimation, the annual number of patients meeting the criteria is over 170.

Inclusion criteria:

1. Scheduled to undergo RALRP or RARC, with an estimated duration of over 3 h
2. American Society of Anesthesiologists’ (ASA) classification of I or II
3. Age is between 18 and 80 years
4. Preoperative troponin T level is normal

Exclusion criteria:

1. Patients with preoperative cardiopulmonary dysfunction who cannot undergo prolonged surgeries in the Trendelenburg position and/or those with severe pulmonary dysfunction with a New York Heart Association (NYHA) classification of III–IV
2. Body Mass Index >30 and weight <18 kg
3. Any intraoperative situation as follows: (1) any cause to cancel the operation or change robot-assisted urological surgery to open surgery, (2) intraoperative cardiovascular accidents

### Study setting

This clinical trial will be conducted in the Huadong Hospital Affiliated to Fudan University and the Ruijin Hospital Shanghai Jiao Tong University School of Medicine, which are tertiary referral centers in Shanghai, China.

### Study group

This study will consist of two arms, the low-pressure pneumoperitoneum group (LP) and the standard-pressure pneumoperitoneum group (SP). The estimated number of patients hospitalized for prolonged robot-assisted urological surgery is 140 in each group. The IAP for the LP group will be held at 7–10 mmHg, while it will be held at 12–16 mmHg for the SP group, both insufflated at low speed (2 L/min), during the robot-assisted urological surgery. The CO_2_ pneumoperitoneum will be administrated using automatic insufflators (Karl Storz, Tuttlingen, Germany).

### Study time

This will be from 1 January 2016 to 31 December 2017.

### Interventions

Clinical monitoring throughout the surgery, including electrocardiography, arterial oxygen saturation/end-tidal carbon dioxide (SpO_2_/ETCO_2_) and invasive measurements of blood pressure will be exerted on all the subjects involved. All subjects will be premedicated with midazolam 2–4 mg administered intravenously after peripheral intravenous (IV) access has been established, following which a central venous catheter will cannulate the right jugular vein. General anesthetic induction will be performed with lidocaine (1 mg/kg), sufentanil (0.3 μg/kg), propofol (4 μg/mL, plasma concentration regulated by targeted controlled infusion (TCI)) and rocuronium (0.6 mg/kg) administered intravenously in sequence. Endotracheal intubation will be performed after muscle relaxation is adequate (TOF is 0). Two to three percent (1.0–1.5 MAC) sevoflurane in a 50:50 (v/v) oxygen-air mixture (1 L/min) and remifentanil (0.1–0.3 μg/kg/min) will be used for anesthetic maintenance until the end of surgery. To achieve adequate analgesia, sufentanil (0.15 μg/kg) will be administered additionally at the time of incision and suture, respectively. To maintain appropriate neuromuscular block, cisatracurium is continuously infused at a rate of 1–2 μg/kg/min until 20 min before surgery completion. Depth of anesthesia is monitored with Narcotrend (MonitorTechnik, Bad Bramstedt, Germany) and the ideal value is 37–64.

MAP within ±30 % of baseline is maintained by using phenylephrine or ephedrine when necessary. Colloids (Voluven, or succinylated gelatin for patients with renal dysfunction) and Ringer’s solution are continuously infused at a ratio of 1:3 to replace fluid losses (from blood loss, urine, respiration and perspiration). When it is imperative, blood products are transfused to maintain physiological balance.

The patients will be mechanically ventilated using volume-controlled ventilation mode and a protective lung ventilation strategy. To maintain an ETCO_2_ level within 35–55 mmHg, the tidal volume or the respiratory rate will be adjusted appropriately.

Patients will be extubated in the Postanesthesia Care Unit (PACU). This action allows the depth of anesthesia to be maintained at a constant level until the last measurement is completed. A patient-controlled intravenous analgesia (PCIA) pump with a concentration of 1.5 mg/mL flurbiprofen axetil injection will be used for all patients postoperatively.

During the surgery, a Trendelenburg position of 30° will be applied to all the subjects involved. The position will be adjusted by using the in-built Compass app for the iPhone 6S (Additional file 1: Figure S1. Apple Inc., Cupertino, CA, USA) that is placed on the table.

Hemodynamic indexes, including heart rate, MAP, central venous pressure (CVP), stroke volume (SV), stroke volume variation (SVV) and cardiac output will be measured using the FloTrac system (Edwards Lifesciences LLC, Irvine, CA, USA).

### Primary endpoint

The troponin T level is set as the primary endpoint for this trial to evaluate myocardial injury, and this will be measured for each patient who will undergo prolonged robot-assisted urologic surgery at the following four time points: Ta (2 to 3 h after surgery), Tb (6 h after surgery), Tc (12 h after surgery) and Td (24 h after surgery) using the fifth-generation high-sensitivity TnT assay.

### Secondary endpoints

Nt-proBNP levels will be measured and recorded at the same time points as for TnT, by the Cobas e 411 analyzer (Roche Diagnostics, Co., Ltd., Mannheim, Germany). Hemodynamic indexes, including heart rate, MAP, CVP, stroke volume (SV), stroke volume variation (SVV) and cardiac output will be measured at the five time points: T1 (5 min after induction), T2 (5 min after CO_2_ pneumoperitoneum is established), T3 (5 min after positioning in the Trendelenburg position), T4 (45 min after Trendelenburg positioning) and T5 (5 min after returning to the horizontal supine position). Respiratory parameters (SpO_2_, ETCO_2_, plateau pressure (PPlat) and tidal volume (Vtide)) will be recorded at all five time points mentioned above.

### Sample size calculation

Based on the summary statistics for the troponin T (TnT) values [29, 32], we use a peak TnT level of 0.01 ng/mL or less as the reference group, and to compute the sample sizes. With 140 patients in each of the two treatment groups, the power will be at least 0.70 to detect an increment of 80 % of patients with a TnT level of 0.02 or higher. The total sample size will be 280, which will ensure the detection of differences of certain pre and perioperative respiratory parameter levels (with power of at least 0.85) [33]. The number of enrolled patients in each center is 140, and the allocation ratio for the two groups is 1:1.

### Randomization and blinding

Patients will be recruited in two hospitals: Huadong Hospital Affiliated to Fudan University and Ruijin Hospital Shanghai Jiao Tong University School of Medicine. Patients who meet the eligibility criteria will be randomly assigned to the two treatment groups: SP or LP. The randomization plans will be implemented using statistical software R, and will be stored in an online database. After a clinical evaluation, the surgeon will register the patient using a unique ID assigned to the patient and used to select a random assignment from the database. The physicians will be aware of the group to which the patients are allocated while the patients will not.

### Data collection and management

The data will be collected according to the primary and secondary endpoints at the time points, and using the methods, described above. All the data will be deposited safely in the in-built server in Huadong Hospital with full confidentiality. Participants in this study will be referred to by a code which differs from their true name. Procedures for data management will be approved by the trial manager and other clinicians before the first participant is enrolled.

### Statistical analysis

The impact of prolonged LPP and SPP on myocardial injury after robot-assisted urological surgery will be evaluated by comparing the changes in the patients with TnT levels of 0.01 or lower. A *t* test or a Fisher’s exact test will be performed as appropriate. The homogeneity of the data collected from the two hospitals will be assessed using the Mantel-Haenszel test. The impact will further be investigated by varying the TnT cutoff, and by examining the effects of CAD and its interaction effect with the main treatment (SP/LP); some other covariates will be justified using a logistic regression model. The distributions of the TnT levels measured at different times will be estimated and the distributional differences will be evaluated between the patients treated with LP and SP, respectively. In addition, the association between the TnT levels measured at T0, T1, T2, T3 and T4 (the peak TnT levels measured at 24 h after surgery) will be assessed. Receiving Operating Characteristic (ROC) curves will be used to assess the value of using the high-sensitivity TnT assays for early detection of elevated TnT levels among patients treated with LP and SP, respectively. The impact of LPP and SPP on pulmonary mechanics and oxidative stress at difference time points will be evaluated using regression analysis, with the effects of CAD and other covariates justified. A statistical analysis plan will be drafted and approved before unlocking the dataset.

## Discussion

Prolonged robot-assisted urological surgery in the Trendelenburg position may include several risk factors that can affect cardiopulmonary physiological status. Our study is designed to investigate the effects of different IAPs on myocardial injury. Physiological changes caused by CO_2_ pneumoperitoneum are complicated, may consist of an intra-abdominal mechanical compressive effect, neurohumoral responses and changes induced by the systemic absorption of CO_2_ [34]. Although previous studies have focused on hemodynamic performance during prolonged robot-assisted surgeries in the Trendelenburg position, few have paid close attention to potential subclinical myocardial injury after different pneumoperitoneal pressures, which may be highly relevant to postoperative mortality.

To our knowledge, the adverse effects caused by utilizing pneumoperitoneum include mechanical compression effects: systemic vascular resistance (SVR), mean arterial pressure (MAP), cerebral blood flow (CBF) and intracranial pressure (ICP) will be increased [35, 36]. On the other hand, renal blood flow (RBF), portal blood flow, splanchnic blood flow and pulmonary compliance will be decreased [37–39].

Additionally, significant hypercapnia and acidosis may occur due to CO_2_ absorption. Hypercapnia may cause a decrease in myocardial contractility. Vasopressin, renin, aldosterone and catecholamine levels also increase sharply after CO_2_ pneumoperitoneum is established [40], with their consequent metabolic and hemodynamic effects.

The Trendelenburg position required by the prolonged robot-assisted urological surgery may cause increased venous return and decreased lung compliance, thus affecting cardiopulmonary function [25, 28, 29]. It commonly causes decreased functional residual capacity (FRC) and decreased pulmonary compliance, increased pulmonary wedge pressure, increased cardiac volume, and normalizing of cardiac afterload while increasing preload. To rule out the inaccuracies caused by changing the angle of the Trendelenburg position, a method to guarantee a fixed angle (30° in this study) is essential. In this trial, we employ a smartphone app to ensure achieving a constant angle for the Trendelenburg position. Once calibrated, the same smartphone will be used for all the patients enrolled. This makes the angle adjustment more convenient and the outcomes more reliable.

Other rare complications of pneumoperitoneum include pneumothorax, inadvertent gas embolism, vascular injury and inadvertent extraperitoneal insufflation.

As described above, the pneumoperitoneal physiology in the Trendelenburg position is intricate. We aim to protect the patients from injury by careful preoperative examination, so that undiagnosed conditions can be identified and pretreated. Standard intraoperative monitoring will be used for all patients, and invasive hemodynamic monitoring (Vigileo with the FloTrac system) will be used to monitor the cardiovascular response to pneumoperitoneum and position changes. ETCO_2_ and airway pressure are commonly assessed to assure adequacy of ventilation and blood gas analysis will be conducted when necessary. Other rare complications will be appropriately treated as soon as they are detected.

Data attrition of the clinical trial would affect the validity of our results markedly. In this study, approaches will be adopted to minimize any impact this may bring. The designed follow-up period of this study is only 24 h post surgery, so it is unlikely that the patients who meet the inclusion criteria would drop out, which is confirmed by the ongoing preliminary trial. Besides, we have the capability for a rapid response to any perioperative emergency events. If attrition does occur, we will carefully discuss the reasons and estimate the impact on the trial. If it is not a source of bias we will employ the complete-case method, where the missing data will be excluded from the analysis. As we have a population large enough to complete the trial in the designated period, this is believed to be the simplest and most efficient method for our study.

The main limitation of this trial is the inevitable bias in age and sex distribution of the enrolled patients. Although the age range in the inclusion criteria is set at 18–80 years, most patients requiring urological surgery, especially resection, are middle-aged and elderly men. Thus the conclusion drawn by this study might be not applicable to female patients and cases in different age groups.

As the trial designed in this study is bicenter, the bias caused by the differences in execution between centers and patient preference of hospital selection might be a challenge. Both the hospitals participating in this trial are tertiary referral centers in Shanghai, but located over 20 km apart and there is an existing long-term collaboration between the two hospitals, which is the basis of this study. From the past years’ patient baseline data, there is no significant competitive recruitment or imbalance between the two hospitals. Thus it could be supposed that the potential bias of those enrolled would not markedly affect the outcome of this study. However, to eliminate any bias caused by variability in anesthesiologists’ abilities, regular meetings and training for clinicians participating in this clinical trial will be ongoing and continued into the future.

## Trial status

Inclusion of eligible candidates in the study has not yet started; enrollment is expected to start in January 2016.

## Additional file

Additional file 1: Figure S1. Schematic diagram for placing the operating table into its correct position preoperatively (30° Trendelenburg position in this study) using a smartphone. (PDF 14017 kb)

## Abbreviations

IAP: Intra-abdominal pressure
LPP: Low-pressure pneumoperitoneum
QTcd: Corrected QT dispersion
RALRP: Robot-assisted laparoscopic radical prostatectomy
RARC: Robot-assisted radical cystectomy
SPP: Standard-pressure pneumoperitoneum
