# Supplementary material for: Comparison of the impact of prolonged low-pressure and standard-pressure pneumoperitoneum on myocardial injury after robot-assisted surgery in the Trendelenburg position: study protocol for a randomized controlled trial
Source: Trials. 2016 Oct 10;17:488. doi: 10.1186/s13063-016-1609-5 (PMC5057242; doi:10.1186/s13063-016-1609-5)
Supplement: Additional file 1: Figure S1. — Schematic diagram for placing the operating table into its correct position preoperatively (30° Trendelenburg position in this study) using a smartphone. (PDF 14017 kb) [file 13063_2016_1609_MOESM1_ESM.pdf]

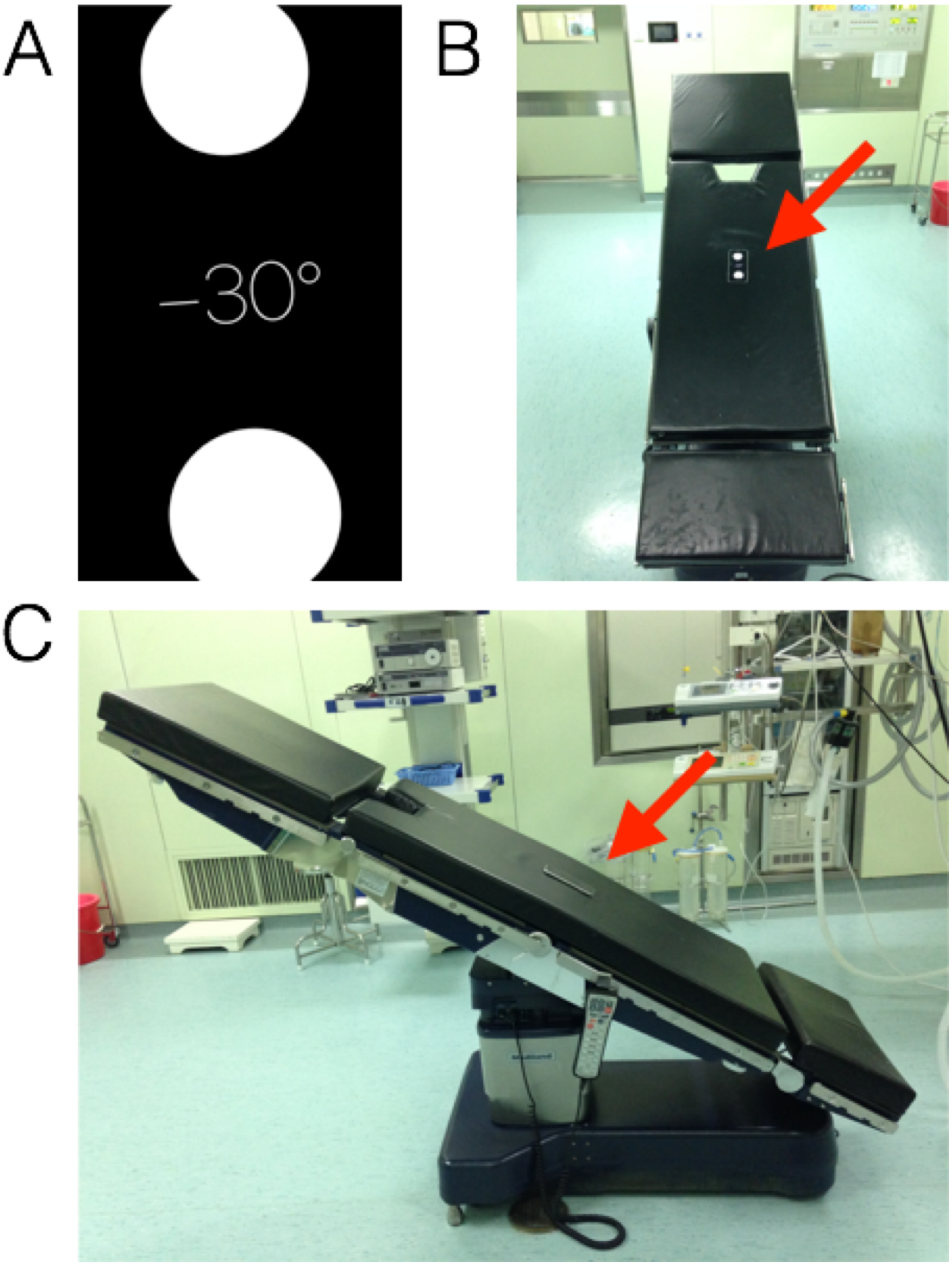

Figure S1. Schematic diagram for place the operating table into correct position (30° Trendelenburg position in this study) with smartphone, before surgery. A. snapshot of the in-built snapshot app of iPhone 6S; B&C, adjust the operating table to a certain angle with a smartphone, before surgery. The position of the smartphone are indicated by red arrow.
